# Spatial models with covariates improve estimates of peat depth in blanket peatlands

**DOI:** 10.1371/journal.pone.0202691

**Published:** 2018-09-07

**Authors:** Dylan M. Young, Lauren E. Parry, Duncan Lee, Surajit Ray

**Affiliations:** 1 School of Geography, University of Leeds, Leeds, United Kingdom; 2 School of Interdisciplinary Studies, University of Glasgow, Glasgow, United Kingdom; 3 School of Mathematics and Statistics, University of Glasgow, Glasgow, United Kingdom; The University of Sydney, AUSTRALIA

## Abstract

Peatlands are spatially heterogeneous ecosystems that develop due to a complex set of autogenic physical and biogeochemical processes and allogenic factors such as the climate and topography. They are significant stocks of global soil carbon, and therefore predicting the depth of peatlands is an important part of establishing an accurate assessment of their magnitude. Yet there have been few attempts to account for both internal and external processes when predicting the depth of peatlands. Using blanket peatlands in Great Britain as a case study, we compare a linear and geostatistical (spatial) model and several sets of covariates applicable for peatlands around the world that have developed over hilly or undulating terrain. We hypothesized that the spatial model would act as a proxy for the autogenic processes in peatlands that can mediate the accumulation of peat on plateaus or shallow slopes. Our findings show that the spatial model performs better than the linear model in all cases—root mean square errors (RMSE) are lower, and 95% prediction intervals are narrower. In support of our hypothesis, the spatial model also better predicts the deeper areas of peat, and we show that its predictive performance in areas of deep peat is dependent on depth observations being spatially autocorrelated. Where they are not, the spatial model performs only slightly better than the linear model. As a result, we recommend that practitioners carrying out depth surveys fully account for the variation of topographic features in prediction locations, and that sampling approach adopted enables observations to be spatially autocorrelated.

## Introduction

Peatlands are globally significant stores of soil carbon, are important for biodiversity, and provide many important ecosystem services such as water regulation, and the provision of food, fibre, and fuel [1,2]. Peatlands develop due to the complex interactions between external forcing and internal biogeochemical processes [3,4] when poorly decomposed plant litter accumulates over millennia in waterlogged conditions. Although external factors such as climate, geomorphology, and the influence of humans play important roles in the initiation, expansion, and fate of peatland carbon (C) [5], their impact can be mediated by internal negative feedback mechanisms [6,7]. This combination of allogenic factors and autogenic feedbacks results in heterogeneous peat accumulation across a peatland [8,9] which make it challenging to assess peat depth over large areas [10]. Blanket peatlands are hyperoceanic ombrotrophic bogs that develop over hilly or undulating terrain covering large areas including all but the steepest slopes. They are found in Europe (Great Britain, Iceland, Ireland, Norway), North America (Labrador, Newfoundland, Alaska), South America (Falkland Islands, Patagonia, Ecuador, Columbia), Asia (e.g. Kamchatka), and Australasia (Tasmania, New Zealand). Similar peatland types (condensation mires), occur in the European Alps and Ruwenzori Mountains in Uganda [11] Because the accumulation of peat in blanket peatlands can occur over varying terrain, allogenic, particularly topographic factors, are perhaps more influential on peat accumulation than on any other peatland type [12,13].

Since the shift in perception that recognizes the wide range of ecosystem services that peatlands provide to societies as a whole rather than just environments of production [14] and the concern that climate change may further destabilize peatland carbon stores [11,15] there has been a widespread drive to conserve and restore peatlands. This drive has been encompassed by global programs, such as Natura 2000 in Europe, which aim to improve biodiversity and ecosystem service provision by restoring 15% of degraded ecosystems by 2020 [16]. Therefore, in order to make decisions about restoration at national, landscape, and catchment scales, policy makers, scientists, and land managers need accurate maps of peatland extent and depth, and ready access to models for predicting peat depth. As a result, improving the large-scale assessment of peat depth is a high priority.

At present manual probing with metal rods or geophysical techniques (airborne and ground based) are used to measure peat depth. However, the spatial resolution, sampling strategy, and quality of the data produced varies considerably between techniques and their application [17]. Manual probing is cheap, technically simple, and due to its portability is able to provide depth measurements at a landscape scale: however, it is time consuming and labour intensive (often the job of volunteers). Conversely, geophysical measurements require specialist equipment and technical ability, and provide depth measurements at a high resolution across small areas [18]. Although geophysical surveys can provide a useful insight into peatland morphology, most peat depth data are gathered using manually probed depth measurements due to the need for a catchment to landscape scale understanding of peat depth variability [18]. However, due to the discrete nature and low spatial resolution of manual probing measurements interpolation must be used to produce maps of peat depth and morphology. As with all digital soil mapping (DSM) challenges, selecting an appropriate interpolation methodology and sampling approach is vital in order to ensure the data gathered can be used effectively and to ensure that representative maps of peat depth are produced [19–23].

DSM incorporates (1) soil, environmental, and spatial inputs; (2) the models that couple the soil attribute of interest and covarying environmental or other soil attributes; and (3) maps of the predicted attribute and its associated uncertainties (see [20] for a review of DSM). As a branch of soil science, DSM studies have demonstrated that topographic variables, spatial position, co-located and neighbouring soil parameters, and environmental covariates can be used to predict the spatial distribution of a soil attribute of interest [19,21]. And although there are important differences between the formation of peat and that of other soils (such as the weathering of parent material in the case of mineral soils), many of the same factors apply (e.g. climate, organisms, spatial position, and relief).

Whilst DSM has been used widely to predict the attributes of mineral soils [20,21], there have been few studies that employ DSM to predict the spatial distribution of peat attributes. Mapping studies of peatlands often predict peat depth as the sole objective or they incorporate peat depth and/or other attributes to estimate soil organic carbon (SOC) stocks [24]. Several of these have used geostatistical methods without covariates, e.g. [25–27], to take into account the spatial dependence of depth measurements, and others have used linear models that do not account for the spatial structure in their data but incorporate environmental covariates (e.g. slope, elevation, and vegetation), e.g. [10,28]. More recently, [29] combined geostatistics without covariates to predict peat depth and multiple linear regression to predict the distribution of other peat attributes such as bulk density and SOC; an updated peat depth map of the Netherlands was produced by [30] by using geostatistics with covarying environmental parameters in a two-step process; and, [31–33] compared machine learning algorithms that coupled field and laboratory measurements with covarying remotely sensed environmental datasets to predict peat depth and/or SOC.

The more recent studies noted above been carried out on raised bogs or permafrost peatlands [31–33]. However, in these peatland types, topographic variables such as slope do not drive the accumulation of peat to the same extent in blanket peatlands. Both [10,28] showed that statistically significant regression relationships exist between peat depth, slope, and elevation. The effect of slope on peat accumulation in blanket peatlands can be seen in field-based observational studies, e.g. [9]. Whereas [31] found that slope did not usefully covary with peat depth in a tropical raised bog. Previous studies on blanket peatlands have either used linear models with covariates [10,28] or geostatistical methods without covariates [27]: the reported performance of these two approaches suggests that improvement should be possible. There have not been any attempts to use geostatistical models with covariates to predict depth in blanket peatlands, or to compare them with identically configured linear models so that the effect of spatial dependence in the observed data can be assessed. Whereas environmental covariates represent the allogenic drivers of peat accumulation, geostatistical models can be seen as a simplified way of representing the autogenic, or internally regulated, drivers without the need to use process-based peatland development models, e.g. [34].

Because blanket peatlands are characterised by both slopes and plateaus, they are well suited to provide a useful insight into combining allogenic and autogenic drivers in an interpolation approach for peat depth mapping. Blanket peatlands in Great Britain and Ireland are the most common peatland type [35] but many have been degraded because of natural processes such as gully erosion [36] the indirect impact of industrialization, and through management for forestry, fuel, agriculture, and game [17] which has deepened water tables and altered vegetation composition [14,17]. Consequently, there is a strong drive by conservationists and peatland managers to develop accurate maps of peat depth at appropriate spatial scales to inform restoration programs in these areas.

Over the past decade land managers and peat restoration projects in the UK and Ireland have invested a considerable amount of resource into gathering blanket peat depth datasets. But although the sampling approach used is known to effect model performance [21–23], a design based on a grid is often used, an approach that has been widely used in the mapping of other soil types [37]. As with all spatially referenced data, peat depth measurements are likely to be spatially autocorrelated (see [38]), meaning that the closer two depth measurements are, the more similar they are likely to be. A proportion of this spatial correlation (smoothness) will be explained by the available covariate factors, but due to the presence of unknown or unmeasured factors that themselves are spatially smooth, some of this correlation will manifest itself in the residuals from any linear model. Therefore, the independent errors assumption underpinning simple linear models is inappropriate, resulting in poorer predictive performance and uncertainty quantification [39]. In this study we build upon previous approaches for modelling blanket peatland depth by fitting and comprehensively evaluating a geostatistical (spatial) modelling approach that, (1) represents both autogenic mechanisms and allogenic drivers of peat accumulation, and (2) accounts for unmeasured spatial autocorrelation. We assess the effect of two sampling approaches on our model predictions and provide recommendations for future peat depth surveys.

## Methods

### Study site and observation datasets

**Fig 1:**
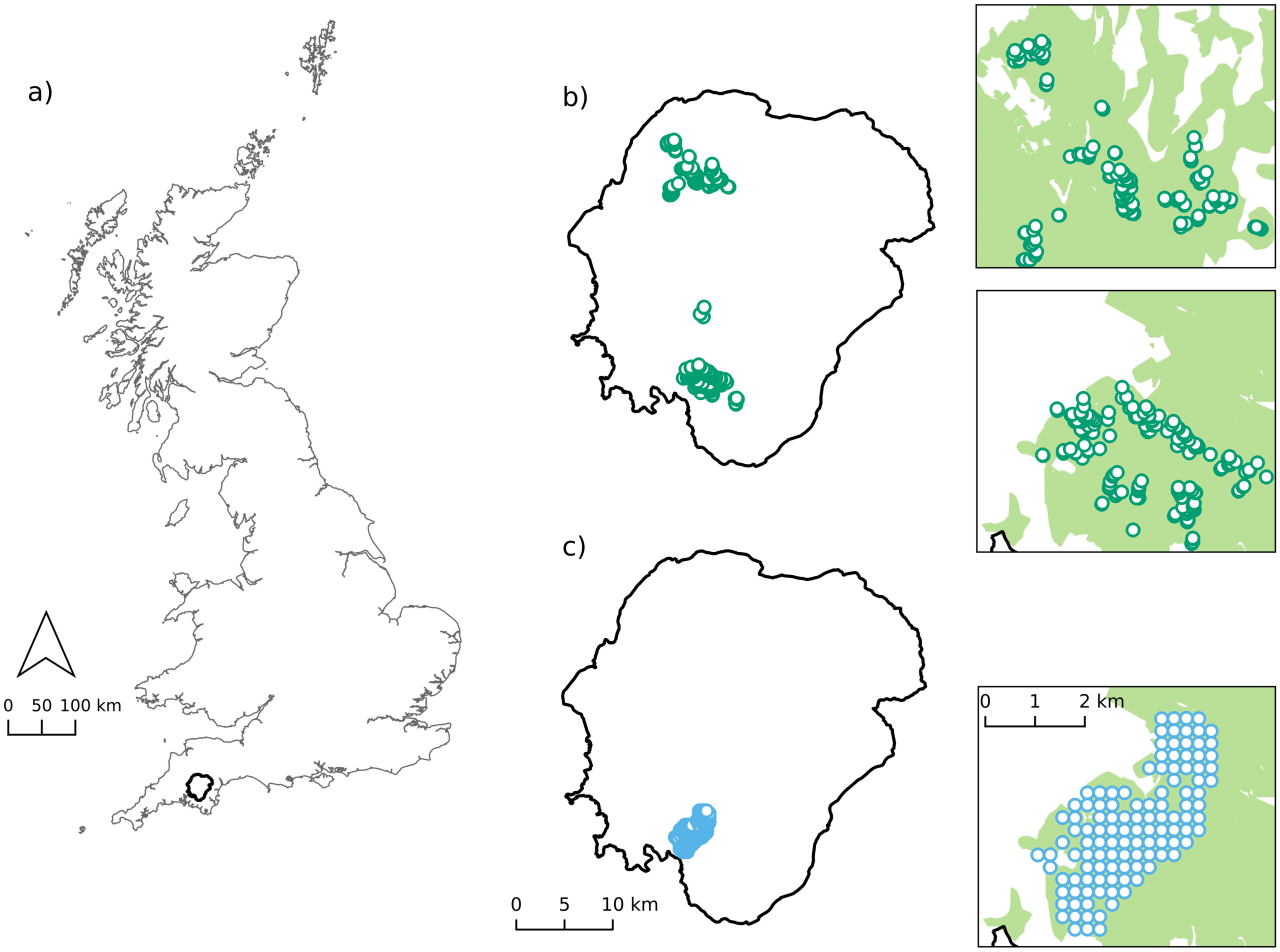
Site and sample locations. (a) Location of Dartmoor National Park (black) in the south west of Great Britain [42,43]. Location of peat depth measurements using (b) stratified sampling observations made at 15–30 m increases in elevation (m asl), and areas of detail for observations in the north and south of the Dartmoor National Park, and (c) a regular grid sampling approach with area of detail. The green shading in areas of detail is blanket peat [44].

We used depth measurements made in blanket peat soils which were a subset of those data reported in [10] from Dartmoor National Park, south-west England (Fig 1a; n = 425; http://dx.doi.org/10.5525/gla.researchdata.604). Two blanket peat soil series—Crowdy and Winter Hill (National Soil Research Institute (NSRI))—cover an area of 122 km^2^, and formed our study site. Dartmoor receives frequent frontal systems with orographic uplift and a high average annual rainfall of 1974 mm [40], this combined with an underlying geology of impermeable granite, has led to the development of an extensive area of hyper-oceanic blanket peatland. The factors controlling blanket peatland development on Dartmoor are similar to other blanket peatlands [41]. The field data used in this study were obtained with permission from National Trust and from Dartmoor National Park Authority.

**Fig 2:**
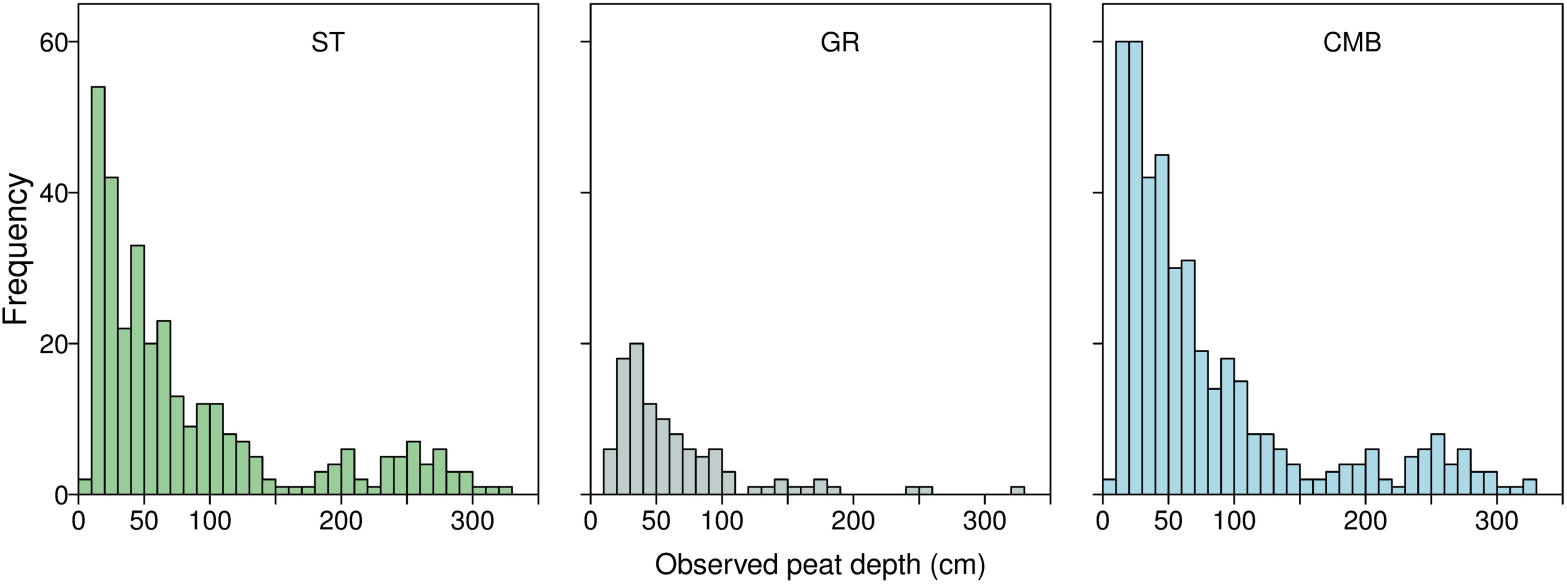
Distribution of peat depth measurements. ST are the observations collected by stratified sampling, GR by gridded sampling, and CMB is the combined ST and GR observations. Observations shown in 10 cm intervals.

All peat depth measurements were recorded using an extendable metal peat depth probe, and five replicates were taken at each sampling point. Points were located in the field using a Trimble Geo XS differential GPS, accurate to 30 cm. A detailed description of the sampling methodology used can be found in [10]. Data within this subset were obtained using two distinct sampling strategies, stratified (ST) and gridded (GR) (Fig 1b and 1c). Dataset ST (Table 1, Fig 2, and S1 Fig) was obtained from both north and south Dartmoor (Fig 1b) and was designed to ensure representative sampling of both slope and elevation [10]. In this dataset, [10] defined a series of spatial units known as ‘carbon unit areas’ which were based on groups of similar soil series and their associated vegetation types. Peat depth was measured at 15–30 m increases in elevation (m asl), and the slope (°) related to each sampling point was derived from a NEXTMap (www.intermap.com) 5 m DEM. Dataset GR (Table 1, Fig 2, and S1 Fig) was the validation dataset used by [10] sampled on a regular grid of 250 m intervals in the south of Dartmoor (Fig 1c). Both datasets included the same vegetation classes (heather moorland, fragmented heather moorland, and grass moorland) and the blanket peat soil associations noted above. We combined datasets ST and GR to create dataset CMB in order to investigate the effect of aggregating different sampling approaches on model predictions. There were 28 locations where peat depth was recorded as 0 cm. We chose to remove these values as they represented measurements taken on mineral soil in gullies or in streams.

**Table 1:** Summary statistics for blanket peat depth datasets.

Observation dataset	Data points	Peat depth (cm)*			Elevation (m asl)		Slope (^o^)	
		Median	Min	Max	Min	Max	Min	Max
ST	319	53.0	5	330	299	601	0.86	22.59
GR	106	46.0	16	330	315	488	1.36	11.64
CMB	425	52.0	5	330	299	601	0.86	22.59
Prediction dataset	DEM nodes				Elevation (m asl)		Slope (°)	
					Min	Max	Min	Max
PR	526655	-	-	-	219	492	0.00	29.17

ST = stratified, GR = gridded, CMB = ST and GR combined, PR = prediction dataset (DEM nodes obtained from a 5 m grid).

* Depth measurements were made to the nearest cm.

### Statistical models

We applied two different statistical models to predict peat depth. The first was a linear model that assumes peat depth measurements are independent after covariate adjustment and is the common modelling approach in this context, e.g. [10]. Because there were no clear non-linear relationships between our depth observations and the selected covariates we chose a linear model for our prediction comparisons. The second was a geostatistical model (hereafter referred to as the spatial model) that allows for the residual (after covariate adjustment) spatial autocorrelation in the data, and thus does not make the unrealistic assumption of independence between spatially close observations [39]. Although geostatistical approaches such as the one used here have been used successfully in the prediction of mineral soil attributes [37], the use of this latter model with covariates to estimate peat depth in blanket peatlands is one of the novel contributions of this paper and we discuss its performance in the next section via a cross-validation approach.

Depth measurements were square-root transformed, as the datasets were non-negative and skewed to the right (Fig 2) and normality was only plausible on this scale. Predictions and model comparison metrics were back transformed to the original scale for model assessment and presentation of results. We predicted peat depth using both linear and spatial models for each dataset (GR, ST, CMB). The models were fitted with six different combinations of the five covariates, and one with no covariates (spatial model only), making a total of 39 model runs. The six covariate combinations were made up from elevation, slope, aspect, vegetation type, and soil (Table 2). To test for the presence of unmeasured spatial autocorrelation, we first fitted a linear model to the datasets using all covariates and computed a semi-variogram for the residuals from this model. The semi-variogram indicated that spatial independence was unlikely, and therefore models that account for spatial autocorrelation should be considered. The purpose of the variogram analysis was to assess if, after full covariate adjustment, the residuals contained any spatial autocorrelation. If not, then a linear model would have sufficed and a spatial model could not have been justified (see [45]).

**Table 2:** Performance metrics for spatial and linear models from 10-fold cross-validation simulations.

	Covariates					RMSE (cm)		Coverage		Interval width (cm)	
Dataset	Elevation	Slope	Aspect	Vegetation	Soil	LM	SM	LM	SM	LM	SM
**ST.C1**	x					12.10	2.61	0.96	0.93	188.02	37.62
**ST.C2**		x				11.14	2.60	0.95	0.93	172.67	35.42
**ST.C3**	x	x				9.22	2.55	0.93	0.93	140.81	35.66
**ST.C4**	x	x	x			8.94	2.54	0.93	0.92	137.78	34.65
**ST.C5**	x	x			x	8.58	2.62	0.92	0.92	132.92	35.30
**ST.C6**	-	-	-	-	-	-	2.58	-	0.93	243.85	37.51
**ST.C7**	x	x	x	x	x	8.53	2.61	0.93	0.93	130.69	34.61
**GR.C1**	x					6.77	5.93	0.95	0.95	106.38	87.90
**GR.C2**		x				5.54	5.02	0.95	0.95	86.92	74.11
**GR.C3**	x	x				5.43	5.03	0.95	0.95	86.02	74.46
**GR.C4**	x	x	x			5.57	5.35	0.95	0.93	86.62	73.80
**GR.C5**	x	x			x	5.50	5.28	0.95	0.94	86.43	73.30
**GR.C6**	-	-	-	-	-	-	6.20	-	0.94	121.56	88.03
**GR.C7**	x	x	x	x	x	5.80	5.76	0.95	0.90	89.81	69.20
**CMB.C1**	x					10.73	3.57	0.95	0.93	165.82	46.92
**CMB.C2**		x				10.18	3.34	0.94	0.93	156.65	43.86
**CMB.C3**	x	x				8.22	3.40	9.93	0.93	126.54	44.18
**CMB.C4**	x	x	x			8.19	3.34	0.93	0.93	125.72	42.73
**CMB.C5**	x	x			x	7.96	3.37	0.93	0.93	122.42	43.88
**CMB.C6**	-	-	-	-	-	-	3.51	-	0.93	213.40	47.15
**CMB.C7**	x	x	x	x	x	7.95	3.37	0.93	0.93	121.42	43.41

ST—stratified sampling, GR—gridded sampling, CMB—combined stratified and gridded observations. C1 to C7 are the sets of covariates used in the predictions. LM = linear model, SM = spatial model.

The spatial model extended the simple linear model by allowing for residual spatial autocorrelation after adjusting for the spatially varying mean, the presence of which was assessed by computing the empirical semi-variogram and corresponding 95% Monte Carlo envelopes based on independence. We also considered a number of spatial autocorrelation approaches including exponential and spherical models [39] and chose the exponential model as it proved to be the best fit to our data in terms of the model evaluation metrics outlined below. All models were fitted via maximum likelihood estimation using R statistical software [46]; and spatial models were implemented using the geoR package [47]. Full details of the models and the software can be found in [39].

The predictive performances of the models were compared using a 10-fold cross-validation approach. Each dataset was split at random into ten subsets of approximately equal size, and each model was fitted to the data from nine of the ten subsets before being used to predict the peat depth at the locations in the tenth subset. This was repeated leaving each of the ten subsets out once, and then the whole process repeated 10 times with different random subsets to ensure the results were robust to the choice of the random subsets. To assess the models’ predictive performance, we averaged the cross validation results over all model runs, and then calculated four metrics. We used; (1) prediction bias and (2) root mean square prediction error (RMSE) to quantify the accuracy and variation in the predictions compared to the measured values; (3) coverage probabilities to calculate the appropriateness of the 95% prediction intervals; (4) the width of the prediction intervals to assess the difference between the upper and lower estimates of peat depth, and (5) Pearson’s Correlation Coefficient (CC) to determine the strength of the linear association between observed and predicted values. Prediction bias quantifies whether on average the predictions are too small or too large (a prediction bias of zero corresponds to the predictions being the correct magnitude on average): we calculated prediction bias as the mean of the predicted value minus the observed value over all locations. And RMSE quantifies the magnitude of the variation between the true and predicted values (lower values indicating better predictive performance). If the prediction intervals are suitable, then coverage probabilities, which are the proportion of the 95% prediction intervals that contain the true values, ideally will be close to 95%.

Based on the results of cross validation, we selected the combination of covariates that over all model runs gave the lowest RMSE, highest CC, and narrowest prediction intervals that had approximately a 95% coverage probability. For this best performing model, we predicted peat depth on a grid with 500 thousand nodes (unmeasured locations) within an area in the south of Dartmoor (Fig 1). The coordinates of unmeasured locations and the values of their covariates were extracted from our 5 m DEM (Table 1; dataset PR). The prediction area contained measured points from both GR and ST sampling methods and allowed us to compare the predictions generated using GR, ST, and CMB datasets.

## Results

### Model predictive performance: Assessment and selection

All spatial models performed better than the corresponding linear models (Table 2); a result that agrees with previous predictions of mineral soil attributes, e.g. [45]. Predictions of depth using the spatial model produced a lower RMSE (the average difference between measured and predicted peat depth) and higher correlation coefficient than the linear model for the same set of covariates across all three datasets (Table 2). For fitted predictions based on peat depths sampled using the stratified method (ST), RMSE varied from 2.54 cm to 2.62 cm for the spatial model, and from 8.53 cm to 15.70 cm for the linear model. However, when we used the dataset obtained by the gridded sampling approach (GR) the differences between models were smaller, which is likely caused by the smaller range of peat depth values (see Fig 2) and the lack of spatial autocorrelation in this dataset. RMSE for the GR dataset ranged from 5.02 cm to 6.20 cm for the spatial model, and from 5.43 cm to 7.70 cm for the linear model. When the datasets were combined (CMB), RMSE varied between 3.34 cm to 3.51 cm for the spatial model, and between 7.95 cm and 13.8 cm for the linear model. The CC for linear models varied between 0.46 to 0.7 for the ST dataset (the CMB dataset results were similar), and 0.34 to 0.54 for the GR dataset (S1 Table): whereas the CC for the spatial models was > 0.9 for the ST dataset, 0.41 to 0.57 for the GR dataset, and 0.88 to 0.89 for the CMB dataset (S1 Table).

Our simulations also show that the average widths of the prediction intervals (difference between upper and lower predictions resulting from the cross-validation exercise) were narrower for all datasets when we applied the spatial model. For the linear model, prediction intervals widths ranged from 130.69 cm to 188.02 cm (ST), 86.02 cm to 121.56 cm (GR), and 121.42 cm to 213.40 cm (CMB). Whilst for the spatial model the prediction intervals were; 34.61 cm to 37.62 cm (ST), 69.20 cm 87.9 cm (GR), and 42.73 cm to 46.92 cm (CMB). Again, of the spatial models, the model using the GR observations performed poorest. Bias and coverage probabilities for linear and spatial models were similar and acceptable. Prediction bias was approximately zero (S1 Table)) showing that the overall predictions of both models are of the correct size (i.e. unbiased, being neither too large or too small). Coverage probabilities ranged from 92% to 96% for the linear model and 92% to 95% for the spatial model (Table 2), identifying that the prediction intervals were of the appropriate width. However, the interval widths produced by the spatial model were narrower across all datasets, suggesting it produced more precise estimates than the linear model.

**Fig 3:**
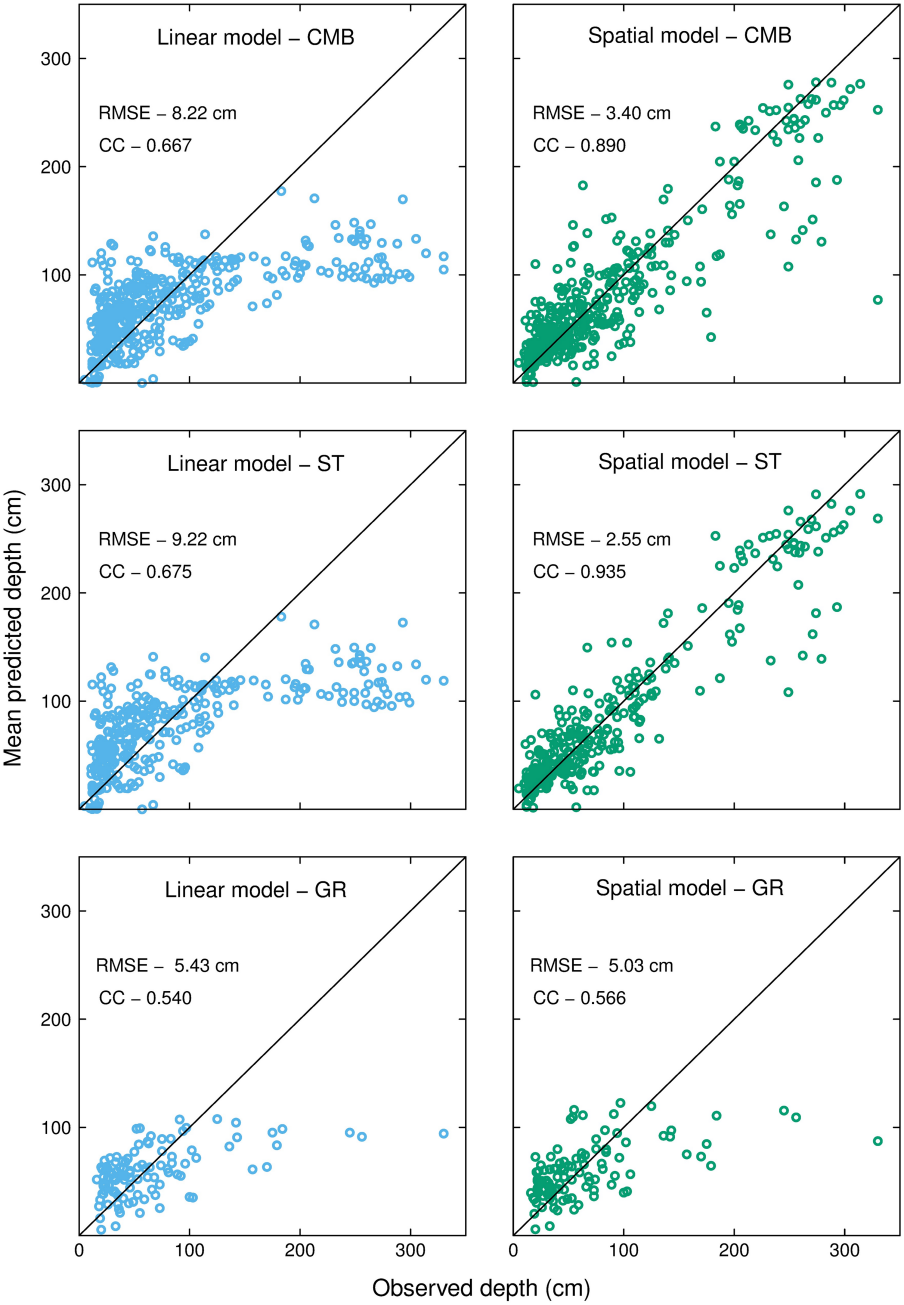
Comparison of mean peat depth predictions and their observed depths between linear and spatial models for the CMB, ST, and GR datasets. Both models were configured with slope (°) and elevation (m asl) as covariates. The predictions were generated using a 10-fold cross-validation approach repeated 10 times for each observation location.

Because our data was collected from different prediction locations, we did not compare model performance between datasets. The results in Table 2 show that the spatial model performs better than the linear model when applied to each dataset; even if only marginally so for the gridded dataset. We also compared the mean depth predictions produced by both linear and spatial models during the cross-validation exercise to their measured locations (i.e. the mean of 10 predictions for each observation location). For this comparison we used each dataset (Fig 3) and included slope (°) and elevation (m asl) as covariates in both model’s formula. Neither model predicted the maximum recorded depth of 330 cm. However, the linear model predicted a maximum mean peat depth of 176.5 cm (Fig 3), whereas the spatial model predicted a maximum mean depth of up to 288.1 cm (Fig 3. The linear model did not predict well the deeper areas of peat across all datasets which we would expect to find on plateaus and in depressions in the landscape (Fig 3). But the spatial model also failed to predict the deeper areas of peat when the GR dataset was used (Fig 3). We suggest that this latter result, and the results shown for the GR dataset in Table 2, is because the GR measurements were taken at 250 m intervals and are spatially independent (S2 Fig), whereas the ST measurements are spatially dependent (S2 Fig). We discuss the implications of the different approaches to depth sampling in the following sections.

### Predictions for unmeasured locations (spatial model)

To select a set of covariates for predicting unmeasured locations, we further assessed the performance of the spatial model by comparing the RMSE and width of the prediction intervals for each of the covariate groups (Table 2; .C1to.C7). Although the RMSE results were similar, the prediction intervals of spatial models that used elevation only (.C1) or no covariates (.C6) performed poorest over the three datasets and we therefore excluded these from our selection process. Of the remaining covariate sets, the differences in RMSE and in the 95% prediction intervals were small (0.06 cm to 0.23 cm, and 1.05 cm to 5.26 cm respectively). Considering our aim to propose a readily accessible approach predict peat depth, we wished to select the most parsimonious model. We therefore chose to combine elevation (m asl) and slope (°) for our predictions at unmeasured locations, as it is trivial to obtain both covariates from a DEM removing the need to collect additional field measurements. Our comparison of predicted and observed depths had shown these two covariates to be effective for predictions across the range of depths in our data. Of these two covariates, a change in slope had the greatest impact on a change in peat depth (S3 Fig) showing the importance of including this covariate in prediction models where peat accumulates on hillslopes. An increase in slope reduces the depth of peat because of increased lateral drainage and a subsequent increase in rapid oxic decomposition [9,13]. Whereas an increase in elevation had a positive effect on peat depth. In our models, elevation was used as a proxy for climate because cooler temperatures at higher elevations can be important for peat accumulation in blanket peatlands [41]. Across the three datasets, a change in slope angle of 1° resulted in a change in peat depth of -0.6 cm to -0.3 cm; and for a change in elevation of 1 m asl, a change in peat depth of 0.011 cm to 0.016 cm (S3 Fig).

**Fig 4:**
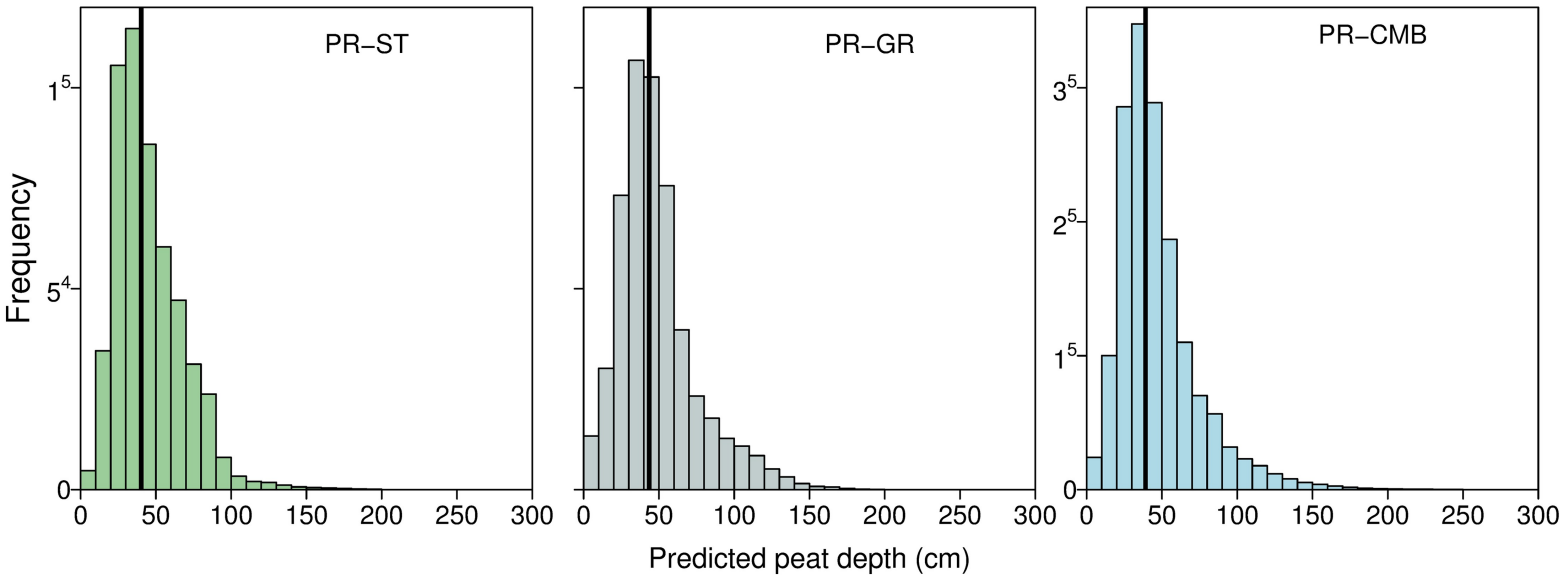
Distribution of peat depth predictions at unmeasured locations. All predictions were obtained using a spatial model that included elevation (m asl) and slope angle (°) as covariates. Dataset PR-ST was based on a stratified sampling approach, dataset PR-GR using a gridded approach, and PR-CMB was obtained using the combined ST and GR observations. The thick vertical line is the median predicted peat depth.

**Fig 5:**
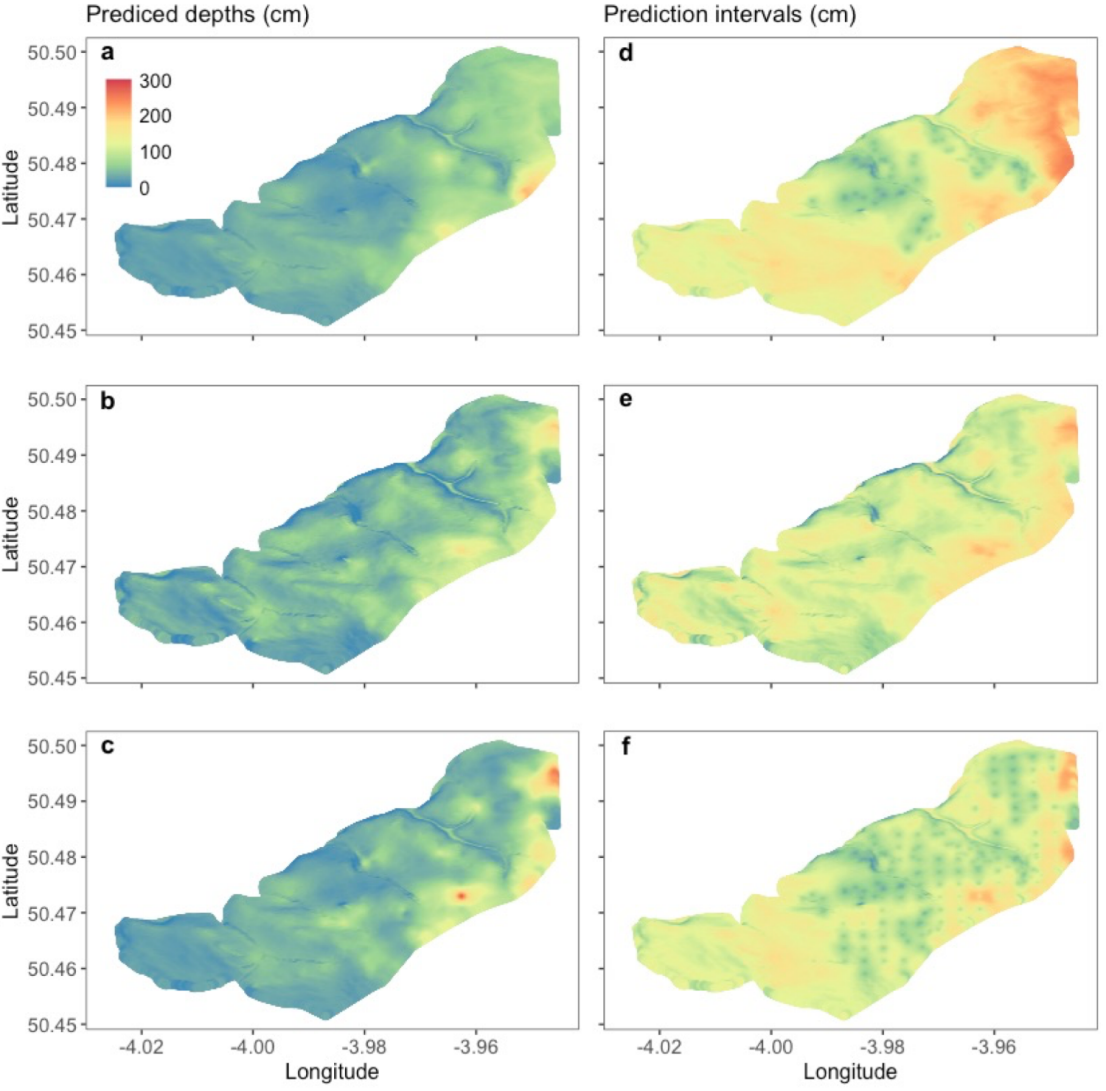
Predicted peat depth for unmeasured locations using the spatial model. (a) Using stratified sampling approach (PR-ST), (b) using gridded sampling approach (PR-GR), and (c) using the combined values from (a) and (b) (PR-CMB). (d-f) 95% prediction interval widths corresponding to the adjacent left-hand side panel. The depth (cm) legend in panel (a) applies to all panels.

Peat depths predicted at unmeasured locations varied between datasets (Fig 4), but median depths were similar; 40.3 cm (PR-ST, stratified), 43.5 cm (PR-GR, gridded), and 39.1 cm (PR-CMB, combined). There were few measurements in deep peat (48 > 200 cm in the complete CMB dataset), and although both sampling approaches included observations of up to 330 cm, the maximum depths predicted by our spatial model was 208.0 cm (PR-ST), and 201.0 cm (PR-GR). Only by combining the datasets (PR-CMB) were depths of up to 291.0 cm predicted. Fig 5 shows the spatial distribution of predicted depths, and their prediction intervals in our test area in the south of Dartmoor. When using the GR dataset, 2534 of the resulting 526655 (~0.5%) predictions were less than zero. In comparison, 23 (< 0.00%) and 63 (0.01%) predictions were less than zero when using datasets ST and CMB respectively. As peat depth cannot be negative, we set each of these values to zero before back transforming our results. This probably occurred because the GR dataset was sampled over a narrower range of both elevation and slope than the ST dataset (Table 1), which resulted in extrapolation of the covariate effects beyond the range of the observations. In addition, Fig 5d shows how the 95% prediction intervals for PR-ST dataset increase as distance from the observations (shown as darker green points) increases: these observations are mainly in two bands across the test area.

## Discussion

### Model performance

This study provides the first predictions of peat depth in blanket peatlands using a model that combines spatial autocorrelation to represent autogenic processes and environmental covariates of important allogenic factors; both of which affect blanket peatland development. Although the accumulation of peat and formation mineral soils differ, our findings agree with those from studies that use geostatistics to predict the attributes of other soil types, e.g. [45,48]. We compared the performance of this geostatistical approach with a linear regression model used in previous studies [10,28] via 10-fold cross-validation using three datasets. Spatial models outperformed the corresponding linear models having both lower RMSE, higher CC, and narrower 95% prediction interval widths (Table 2). We propose that this result is because linear models do not account for autogenic processes that can mediate peat accumulation in peatlands.

Peatland development is a network of complex of interactions between biogeochemical processes, physical processes, geomorphology, the influence of humans, and the climate [5]. Peatland initiation and subsequent development takes place over millennia and is mediated by self-regulating feedback mechanisms (for an example of the range of hydrological feedbacks in northern peatlands see [4]). Therefore, the rate of peat accumulation is not dependent on allogenic covariates alone. The spatial approach used here aims to recognise that peat accumulation in blanket bogs is strongly affected by these feedback mechanisms, as well as by topographic factors such as slope and elevation [10,28,41]. For example there is a negative feedback between hydraulic conductivity, decomposition, and water-table depth [4,34,49]. Deepening water tables lead to increased oxic decomposition which reduces hydraulic conductivity, this processes in turn slows lateral drainage causing water tables to become shallower once more. The distribution of hydraulic conductivity, and other peat properties, can vary both vertically and horizontally [13,50,51], but measurements of these properties are not trivial to obtain and are not therefore widely available. However, we suggest that their influence is ‘wrapped up’ in the spatial autocorrelation of the data in our model.

We also show that where the development of a peatland is affected by topographic factors, spatial models that incorporate these covariates perform better (narrower 95% prediction interval widths) than the geostatistical approaches without covariates used in some previous studies, e.g. [27,52]. It is notable that, the actual combination of covariates included in the spatial model makes little difference to the variation or width of predictions. The model with slope as the single covariate performed similarly to the model that included all covariates, supporting the finding of [10] that, in the case of blanket peatlands hillslopes, slope was the most dominant predictor of depth (S3 Fig). However, we also found that the variation and width of the depths predicted by the linear model were more sensitive to the choice of covariates.

As we show in Fig 3 the spatial model with covariates resulted in useful predictions across the range of peat depths as opposed to the linear model which only performs well in areas of the landscape where slope is the dominant factor in peat accumulation. However, we did not include past climate in our model which is an important factor in peatland development [53] but would be challenging to build in to our models, and make it less likely that our model would be useful for practitioners (although elevation can be seen as a proxy for temperature). Our spatial approach to predicting peat depth demonstrates an improvement over linear regression models because peatland size and shape emerges from the interaction of small-scale processes. However, it is difficult to fully account for this network of interactions unless the processes themselves are simulated in process-based models [3,54]. Although such models exist for peatlands [55,56] they tend to be computationally expensive when run in 3-D over landscape scales, and records of past climate at relevant timescales and measurements of underlying topography are needed. In addition, these models are generally used to investigate the future effect of climate and land use [34,57] which go beyond the predictive boundaries of statistical models based on current observations [58]. The approach used here can therefore provide a useful and practical bridge between statistical approaches that account for both spatial autocorrelation and key covariates, where the aim is to predict past peat accumulation for carbon accounting purposes, and ecosystem models used to understand the effects of novel conditions on future peat accumulation over timescales of decades to centuries.

### Prediction of unmeasured locations

Despite the spatial model with covariates being shown to have the strongest performance of the models used in our study, when we used it to predict peat depth at unknown locations the quality of outputs was not spatially consistent and model performance declined on plateaus where depth predictions were too shallow and widening interval widths were observed (Fig 5). Although a maximum depth measurement of 330 cm was included in both sampled datasets (Table 2), the ST and GR datasets predicted similar maximum depths of 208 cm and 201 cm respectively. And both the ST and GR strategies showed poor performance when predicting known depths in areas of deeper peat which typically occur on shallow slopes (Fig 5). However, the combined dataset (CMB) model produced a maximum predicted depth of 291 cm—closer to the known maximum value. There are a number of reasons why this might be the case; (1) ST observations occur in two bands across the test area (Fig 5d–dark green points), which lead to an increase in the 95% prediction intervals because the observations are being used to predict the depth of distant locations. (2) Although the observations obtained from gridded sampling were distributed more evenly throughout the test area, they were not spatially autocorrelated and model performance is similar to that shown for the model comparisons in Fig 3 (i.e. performance decreases where depth increases) [45]. (3) Few of our observations were made within areas of deep peat (14 > 200 cm in the test area). In these locations, slope is no longer the dominant driver of peat accumulation and autogenic interactions are more important. (4) Combing both datasets (PR-CMB) increases the distribution of points over the test area, and also increases the number spatially autocorrelated points as some of the PR-GR points are located close to PR-ST points. As a result, we found that our spatial model performed best in the test area when we combined both the datasets (CMB) to increase the number and range of observations (Fig 5c and 5f).

The prediction of peat depth across plateaus is a significant challenge and incorporating spatial autocorrelation as a proxy for autogenic processes in these locations was key to our rationale for choosing a spatial approach. The formation of peat deposits across plateau regions may include basins within the underlying topography that infill with peat. And several of these basins may also coalesce into to foci of deep peat connected by younger, shallower deposits as shown by [9] Evidence of this difference between surface and the underlying base material has been reported previously on Dartmoor by [59] who identified an isolated area of deep peat (> 7 m) surrounded by shallower accumulations. Conversely, on steeper slopes where peat depth is typically shallower, peat deposits do not fully infill depressions in underlying geology, leaving the slope-depth relationship intact. Both [10,28] encountered this problem using a linear interpolation approach. Although we have shown that our spatial model is an improvement over a linear model with the same covariates (Table 2 and Fig 3), our work has highlighted that model choice alone is unlikely to resolve the errors associated with peat depth maps in plateau regions if sampling campaigns produce sparse measurements or neglect to include deeper accumulations. Nevertheless, there may be potential to resolve this issue by using an informed approach to sampling strategy that builds on the strengths of the spatial model, and we discuss the possibilities for doing this in the following section.

### Sampling strategy

Although there is substantial interest in gathering probed depth datasets for all peatland types, there is little in common between the spatial approach of the sampling strategies used. For example, [28] used a series of transects, [10] applied a stratified random sampling approach, and [27] used a gridded design. When carrying out spatial interpolation, our results support previous studies who have shown that sampling design can have a considerable impact on the quality and validity of the interpolated mapping produced, e.g. [60]. The choice of sampling strategies is governed and affected by several factors, including the interpolation approach being used, the human resources available, distance between measurements, and site remoteness and accessibility [22].

Often peat depth datasets are created from several sampling campaigns, e.g. [61], and can have multiple sampling strategies integrated within the dataset. Understanding the relative quality of interpolated depth maps using differing sampling strategies would improve the understanding of error when mapping peatland ecosystem services, particularly carbon storage. A large amount of time and effort is employed to gather peat depth measurements and therefore an understanding of how the sampling strategy of future peat depth surveys could be most effectively designed and applied is highly important. Standardising sampling design should improve consistency between datasets and facilitate an understanding of error and the quality of depth predictions across datasets and peatlands.

We found that where observations were not spatially dependant the differences in model performance between sampling strategies was less evident (see [45]), and therefore we can also expect that it will be difficult to predict areas of deep peat even with a spatial model: this is evident from the predictions done with the GR dataset (see Table 2) where observations were made every 250 m. However, whilst the difference in RMSE of both approaches is small (0.1 to 1.5 cm), the difference in prediction intervals is sufficiently large (11.6 to 33.5 cm) to favour the use of the spatial model despite the imitations of the sampling approach. It is possible that closer spacing of a gridded sampling design in plateau areas may negate this issue, but further investigation is required. A further issue with the GR dataset was that it did not incorporate the full range of values of model covariates (Table 1), and a substantial number of the unknown prediction locations had slope angles beyond the maximum value in the GR dataset. Therefore, predictions of the spatial model that used the GR dataset were constrained by this artefact whereas those based on the ST dataset were not, and were typically deeper. Clearly there is the potential to supplement existing datasets with new targeted observations which could improve any estimates of SOC.

However careful consideration also needs to be given to the design of stratified sampling strategies. In our case, the ST dataset did not have enough points (including observations in deep peat) located throughout the extent of the plateau region of the test area (Fig 1). This may mean that although we chose the spatial model to represent autogenic processes, a suitable number of sampling points were not available to sufficiently represent these processes. Alternative approaches to stratify the study area with the aim of improving the range of covariate space sampled, e.g. [23,62], and could be used to enhance existing peatland datasets where the covariate range has not been sufficiently represented.

From these analyses, there is a clear difference between the characteristics and quality of outputs using different sampling strategies. There is strong evidence that whilst the spatial approach has the potential to produce a better set of depth predictions (low RMSE and narrow 95% prediction interval widths), this potential is affected by the design of the sampling strategy to a greater degree than the linear model (which is a poor predictor of deep peat). Therefore, it is important that practitioners and those designing peat depth sampling campaigns in blanket peatlands consider how to maximise the utility of the model when designing their sampling strategy (see for example [22]). Our results indicate that this means there is a need to take account of the targeted site’s topography when designing a sampling strategy, as differences were seen in response to sampling strategy between steep and plateau areas. If the site has a substantial region of plateau we suggest that as many observations as possible are made in these areas when using a stratified approach. We also show that if the area to be surveyed includes hillslopes, the sampling strategy must represent the full spectrum of slopes present.

## Conclusions

Peatlands are globally important stores of carbon and therefore depth predictions are central to estimating their magnitude [63], yet few models incorporate the processes that drive peat accumulation in peatlands that form over both hillslopes and plateaus. We show how a spatial (geostatistical) model can be used as an improvement over linear models and those that are based on a kriging approach without covariates. Our model is the first to estimate peat depth in blanket peatlands that includes both covariates that drive peat accumulation on slopes and spatial autocorrelation as proxy for the autogenic processes that play an important role in moderating peat accumulation on plateaus. However, we have also shown how sampling survey design can limit the effectiveness of the approach used here. We therefore propose that;

1. Spatial models with key covariates are used to predict peat depth. Our model can be implemented with few difficulties as a low-cost approach when coupled with DEMs created in open source GIS.
2. Sampling campaigns use a stratified approach to include the full range of covariates that can be omitted by using gridded designs. And where necessary, existing datasets should be supplemented with new observations targeted at incorporating the range of values for a given covariate.
3. Plateau regions are sampled as extensively as is practical and other information (previous surveys, local knowledge) be used to identify and include areas of deep peat in a survey. If possible, pre-survey site visits should be made to validate these areas.
4. Sampled data is checked to ensure it is spatially autocorrelated, and additional measurements made if necessary.

## Supporting information

S1 Fig Distribution of slope and elevation for the locations of peat depth. ST = stratified dataset, and GR = gridded dataset. Refer to main text for a description of the differences between the two observation datasets.
(JPEG)

S2 Fig Estimated semi-variograms computed from the residuals of three datasets used for peat depth predictions after fitting a linear model of the square root of peat depth. Slope (°) and elevation (m asl) were used as predictors. The dashed lines are upper and lower 95% confidence intervals for the semi-variograms generated under independence using a Monte Carlo approach. (a) Stratified observations (GR), (b) gridded observations (ST), and (c) combined (CMB) gridded and stratified observations.
(TIFF)

S3 Fig Covariate importance for predicting blanket peat depth for the spatial model with slope and elevation as covariates. Point estimates are the effect of a change in one unit of the covariate (slope (°), elevation (m asl)) on peat depth (cm). Horizontal lines are the 95% confidence intervals. ST.C3, GR.C3, and CMB.C3 are the stratified, gridded, and combined datasets and covariate combination (refer also to Table 2 in the main text).
(JPEG)

S1 Table Prediction bias and correlation coefficients for linear and spatial models for covariate groups (Peat depth). (PDF)
