# Supplementary figures and images for: Spatial models with covariates improve estimates of peat depth in blanket peatlands
Source: PLoS One. 2018 Sep 7;13(9):e0202691. doi: 10.1371/journal.pone.0202691 (PMC6128521; doi:10.1371/journal.pone.0202691)

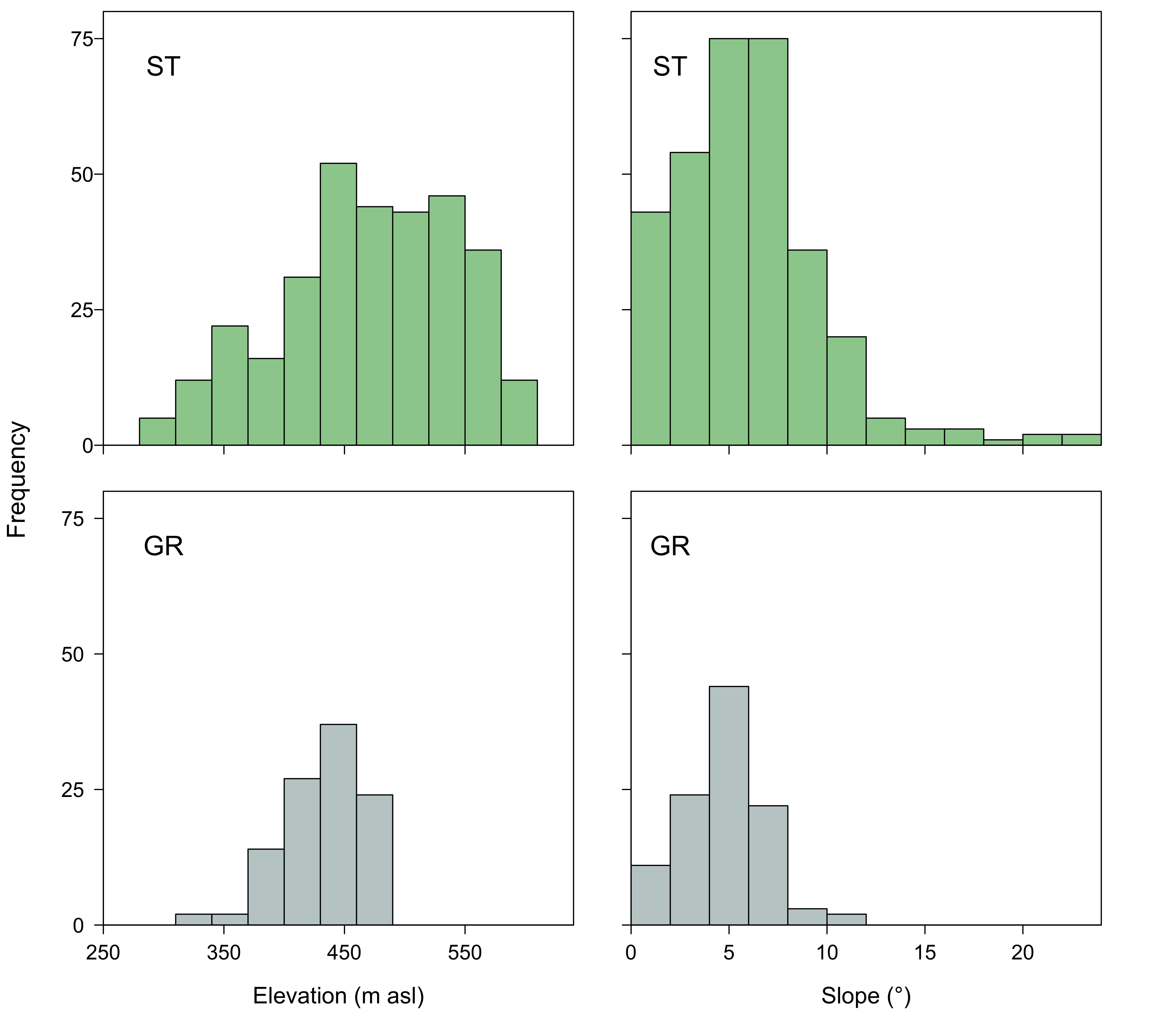

Supplement: S1 Fig — ST = stratified dataset, and GR = gridded dataset. Refer to main text for a description of the differences between the two observation datasets. (JPEG) [file pone.0202691.s001.jpeg]

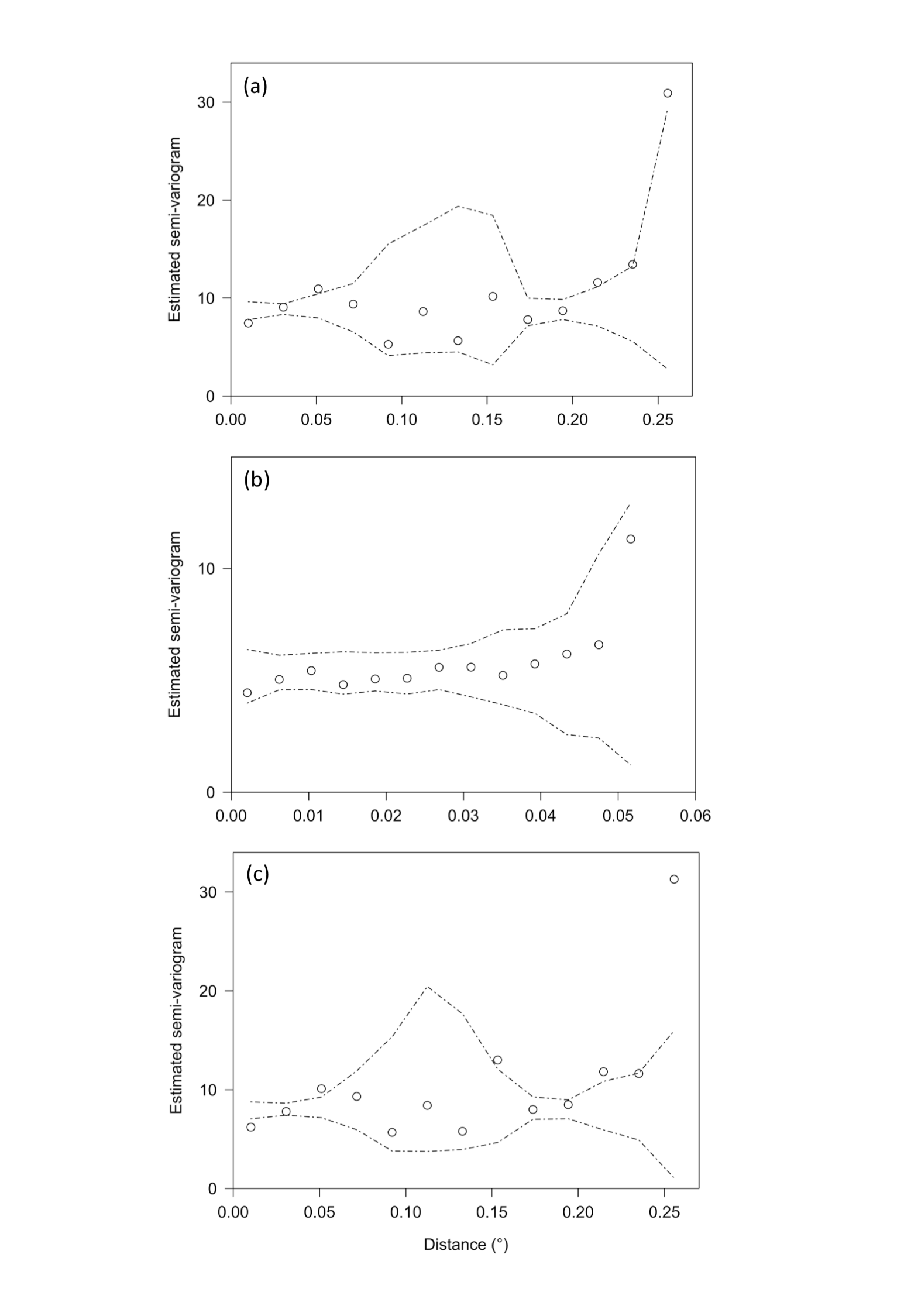

Supplement: S2 Fig — Slope (°) and elevation (m asl) were used as predictors. The dashed lines are upper and lower 95% confidence intervals for the semi-variograms generated under independence using a Monte Carlo approach. (a) Stratified observations (GR), (b) gridded observations (ST), and (c) combined (CMB) gridded and stratified observations. (TIFF) [file pone.0202691.s002.tiff]

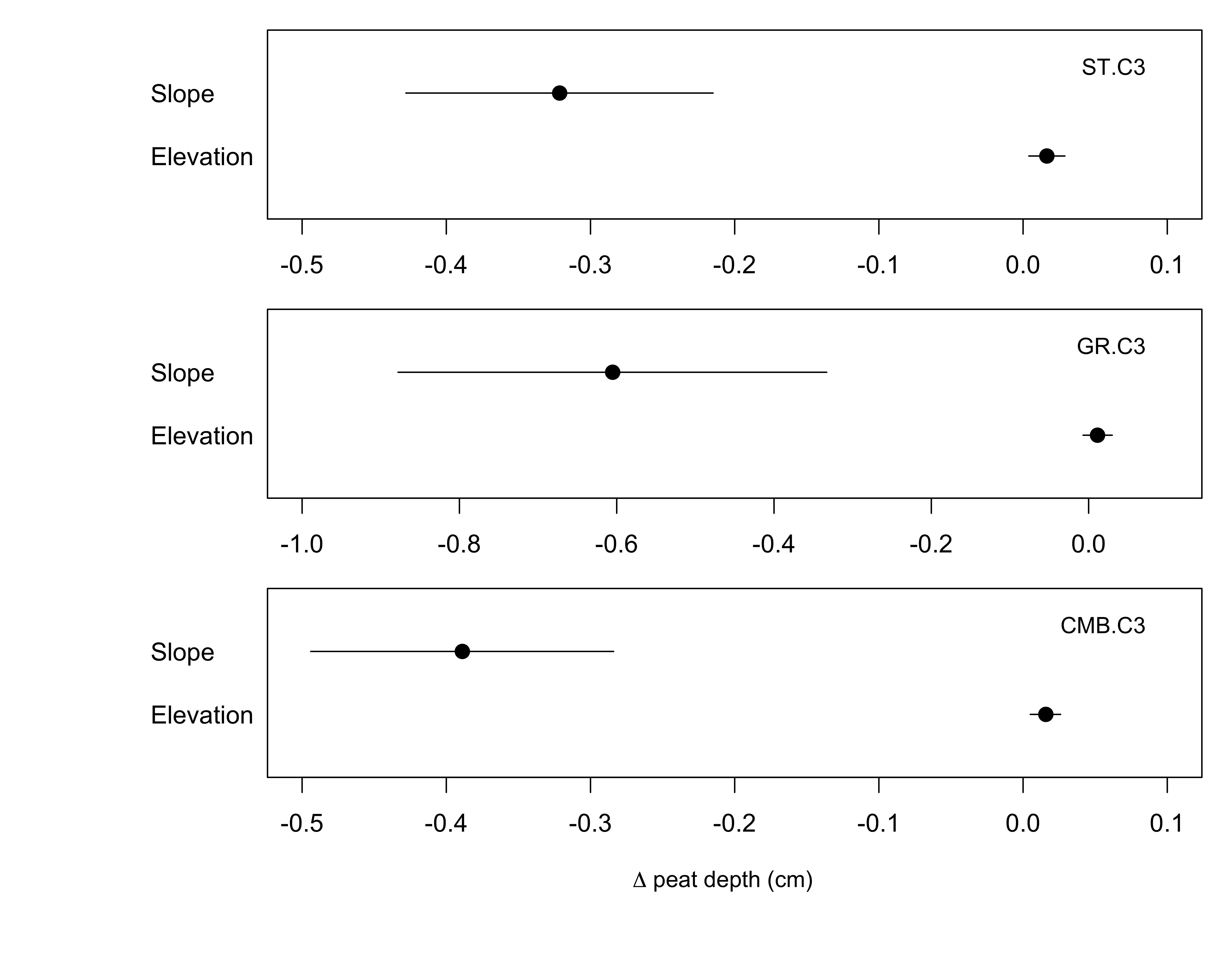

Supplement: S3 Fig — Point estimates are the effect of a change in one unit of the covariate (slope (°), elevation (m asl)) on peat depth (cm). Horizontal lines are the 95% confidence intervals. ST.C3, GR.C3, and CMB.C3 are the stratified, gridded, and combined datasets and covariate combination (refer also to Table 2 in the main text). (JPEG) [file pone.0202691.s003.jpeg]
