# Supplementary material for: Spatial models with covariates improve estimates of peat depth in blanket peatlands
Source: PLoS One. 2018 Sep 7;13(9):e0202691. doi: 10.1371/journal.pone.0202691 (PMC6128521; doi:10.1371/journal.pone.0202691)
Supplement: S1 Table — (PDF) [file pone.0202691.s004.pdf]

S1 Table.

	Covariates								
Dataset	Elevation	Slope	Aspect	Vegetation	Soil	Bias LM	Bias SM	CC LM	CC SM
ST.C1	x					-0.0007	0.0620	0.462	0.931
ST.C2		x				-0.0045	0.0517	0.531	0.934
ST.C3	x	x				-0.0109	0.0477	0.675	0.935
ST.C4	x	x	x			-0.0106	0.0575	0.687	0.936
ST.C5	x	x			x	-0.0071	0.0638	0.687	0.934
ST.C6	-	-	-	-	-	-	0.0770	-	0.931
ST.C7	x	x	x	x	x	-0.0059	0.0481	0.698	0.934
GR.C1	x					-0.0182	-0.0117	0.341	0.457
GR.C2		x				-0.0121	-0.0222	0.524	0.563
GR.C3	x	x				-0.0101	-0.0037	0.540	0.566
GR.C4	x	x	x			-0.0110	-0.0254	0.517	0.530
GR.C5	x	x			x	-0.0062	-0.0052	0.510	0.522
GR.C6	-	-	-	-	-	-	0.0171	-	0.414
GR.C7	x	x	x	x	x	0.0037	0.0021	0.464	0.468
CMB.C1	x					-0.0014	0.0512	0.462	0.883
CMB.C2		x				-0.0062	0.0507	0.503	0.893
CMB.C3	x	x				-0.0046	0.0405	0.667	0.890
CMB.C4	x	x	x			-0.0054	0.0343	0.671	0.891
CMB.C5	x	x			x	-0.0050	0.0502	0.667	0.890
CMB.C6	-	-	-	-	-	-	0.0679	-	0.885
CMB.C7	x	x	x	x	x	-0.0095	0.0386	0.675	0.890

Dataset sampling process: ST = stratified, GR = gridded, CMB = combined. C1-C7 are the covariate groups used. LM = linear model, SM = spatial model. CC = Pearson's Correlation Coefficient.
